# Building an Otoscopic screening prototype tool using deep learning

**DOI:** 10.1186/s40463-019-0389-9

**Published:** 2019-11-26

**Authors:** Devon Livingstone, Aron S. Talai, Justin Chau, Nils D. Forkert

**Affiliations:** 1 Division of Otolaryngology – Head and Neck Surgery, Department of Surgery, University of Calgary, 7th floor, 4448 Front Street SE, Calgary, Alberta T3M 1M4 Canada; 2 Department of Radiology, Hotchkiss Brain Institute, University of Calgary, Calgary, Canada

**Keywords:** Neural network, Machine learning, Automated, Otoscopy, Deep learning, Artificial intelligence

## Abstract

**Background:**

Otologic diseases are often difficult to diagnose accurately for primary care providers. Deep learning methods have been applied with great success in many areas of medicine, often outperforming well trained human observers. The aim of this work was to develop and evaluate an automatic software prototype to identify otologic abnormalities using a deep convolutional neural network.

**Material and methods:**

A database of 734 unique otoscopic images of various ear pathologies, including 63 cerumen impactions, 120 tympanostomy tubes, and 346 normal tympanic membranes were acquired. 80% of the images were used for the training of a convolutional neural network and the remaining 20% were used for algorithm validation. Image augmentation was employed on the training dataset to increase the number of training images. The general network architecture consisted of three convolutional layers plus batch normalization and dropout layers to avoid over fitting.

**Results:**

The validation based on 45 datasets not used for model training revealed that the proposed deep convolutional neural network is capable of identifying and differentiating between normal tympanic membranes, tympanostomy tubes, and cerumen impactions with an overall accuracy of 84.4%.

**Conclusion:**

Our study shows that deep convolutional neural networks hold immense potential as a diagnostic adjunct for otologic disease management.

## Introduction

Access to tertiary care ear, nose, throat (ENT) specialists is extremely limited in many regions outside of major academic centers worldwide, while lengthy waitlists in metropolitan areas impair timely access to quality care. Otologic disease treatment is highly subspecialized, creating diagnostic difficulties for primary care providers, ultimately leading to negative impacts on patients. The burden of initial diagnosis and triage largely falls upon general practitioners and other allied health professionals, who are relatively inaccurate with otologic diagnoses, even for common presentations such as acute otitis media or serous otitis media [1–4]. For example, community pediatricians and general practitioners only have a diagnostic accuracy for acute otitis media of 62% with pneumatic otoscopy [2]. A multi-national study of pediatricians and general practitioners showed that the average diagnostic accuracy for acute otitis media and serous otitis media using video otoscopy is only 51 and 46%, respectively [4]. Otolaryngologists are more accurate, though very far from perfect, with diagnostic accuracies around 74% [4, 5].

Clearly, there is a need for an improved method for screening patients for otologic abnormalities so that otologic complaints can be correlated with relevant otoscopic findings. Previously described methods for automatic otoscopic diagnosis have mostly relied on handcrafted feature extraction techniques such as edge detection or histogram analysis [6, 7]. These approaches are limited to a select number of diagnoses, such as tympanostomy tubes [6] or otitis media [7], and are further limited by poor generalizability when using different imaging techniques.

Recently, deep neural networks have been applied with great success in various areas of medicine, often achieving higher accuracy compared to traditional machine learning techniques and comparable performance to highly trained human specialists [8–11]. Convolutional neural networks (CNNs) are a variation of deep neural networks capable of recognizing features within images. Broadly speaking, CNNs are a type of machine learning tool that are constructed and modelled in a way the mirrors the neural networks found in biological brains. In detail, a CNN can learn to identify the appearance of abnormalities after being trained using labeled normal and abnormal images without the requirement of explicit handcrafting of features to do so. In detail, these features are learnt at the same time as the network is optimized based on the training set. In this context, CNNs are an emerging adjunct to medical diagnosis and there is a large body of scientific literature devoted to their use for numerous medical applications [8, 9, 12, 13]. Common otologic diseases, such as acute otitis media, serous otitis media, and tympanic membrane perforation have unique appearances that might be detectable using deep convolutional neural networks. Despite this high potential for deep convolutional neural networks for diagnosing ear diseases based on digital otoscopic images, this has not been tested so far.

Therefore, the objective of this study was to develop and evaluate a software prototype using a deep convolutional neural network model for identifying abnormalities in otoscopic ear images. Such a computer-aided diagnosis tool based on deep neural networks would facilitate appropriate triaging for subspecialist review and facilitate timely access to accurate otologic diagnosis and treatment.

## Material and methods

### Patients and imaging

Ethical approval for this study was obtained from the Conjoint Health Research and Ethics Board at the University of Calgary, AB, Canada. Otoscopic images were collected prospectively from patients presenting to Alberta Health Services facility outpatient otolaryngology clinics in Calgary, AB, Canada. CellScope [14] smartphone adaptors were utilized to obtain the clinical images. All otologic pathologies and normal healthy tympanic membranes were included in the database.

### Ground truth diagnosis

All images were reviewed in consensus by two experienced ENT specialists (DL & JC) taking into account the diagnosis in the corresponding electronic health care records. Abnormalities included the presence of a myringotomy tube, either in appropriate position in the TM or extruded, cerumen impaction, and a healthy, normal tympanic membrane.

### CNN training, optimization, and testing

The three most common diagnostic categories (normal tympanic membrane, tympanostomy tube, and cerumen impaction) were utilized to train and test the CNN. Image augmentation in the form of rotation and mirroring was employed on the dataset to increase the number of training images in each category to achieve balanced training sets. All images were converted to grayscale before being passed to the network. Table 1 represent the breakdown of datasets in each category. A small sized network, consisting of three convolutional hidden layers, where each layer contained a batch normalization (BN), and a dropout layer with a fixed ratio of 40% was implemented in the TensorFlow frame work in Python.

**Table 1:** Algorithm Performance for Normal, Tube and Cerumen Diagnoses

Diagnosis	Database Images *n*	Post Processing *n*	Algorithm score	Misclassifications	Accuracy %
Normal Tympanic Membrane	346	993	14/15	Myringosclerosis	93.3
Tympanostomy Tube	120	1050	13/15	Cerumen Cerumen	86.7
Cerumen Impaction	63	960	11/15	Normal Normal Normal Normal	73.3
Total	529	3003	38/ 45		84.4

20% of the overall training datasets were used as a validation set in order to optimize the CNN’s hyper-parameters via the so-called babysitting approach [15]. Furthermore, 15 images in each diagnostic category were isolated for testing the algorithm’s performance, yielding a 45-image test set. It is important to note that none of the testing images including augmented versions were part of the training or validation datasets.

## Results

### Diagnosis dataset

**Fig. 1:**
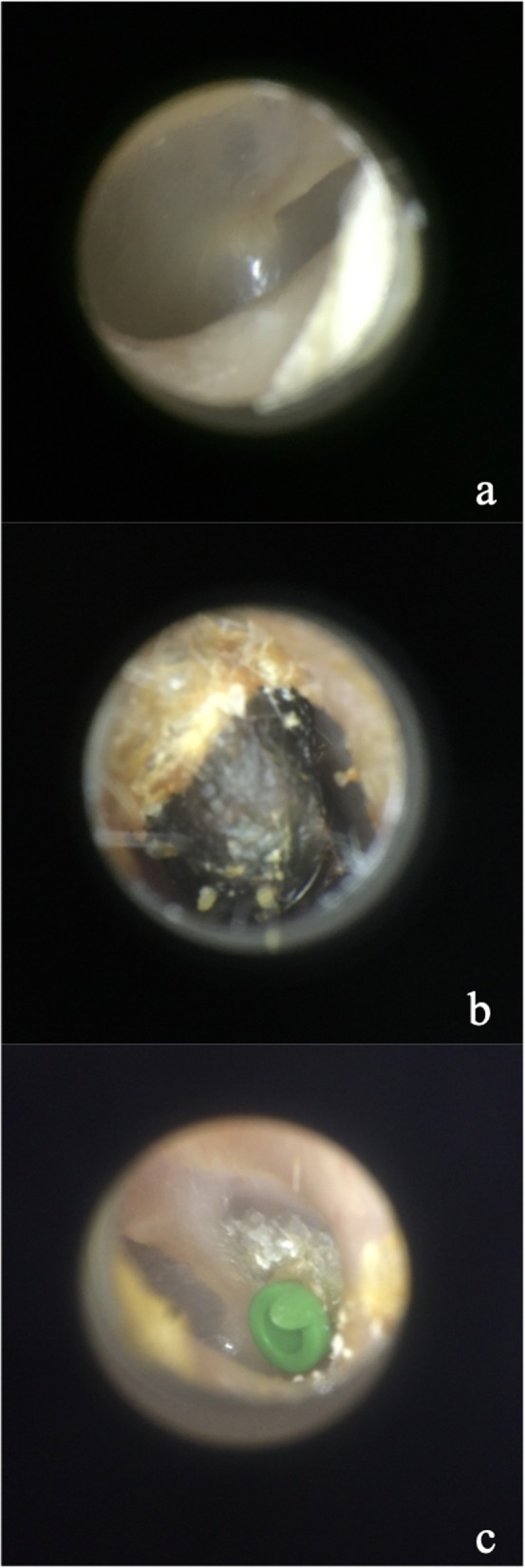
Selection of images obtained using CellScope smartphone attachment **a**) normal **b**) cerumen **c**) tympanostomy tube

Overall, 724 unique otoscopic images of various otologic diagnoses, including 346 normal tympanic membranes, 120 tympanostomy tubes, 63 cerumen impactions, 50 tympanic membranes with myringosclerosis, 44 with otitis externa, and 32 tympanic membranes perforations were acquired and available for this study. While all images were used for algorithm training, the test set was constrained in terms of available diagnoses; only the normal, tympanostomy tube, and cerumen impaction categories were selected for CNN model testing in this work. These categories had the largest number of images, which is required for accurate performance of the CNN. The other categories had insufficient image numbers and were therefore not included in the final test set used, though the algorithm was constructed to allow for diagnosis of all conditions found in the dataset. Figure 1 shows a selection of the study images obtained using the CellScope attachment.

### Algorithm performance

**Fig. 2:**
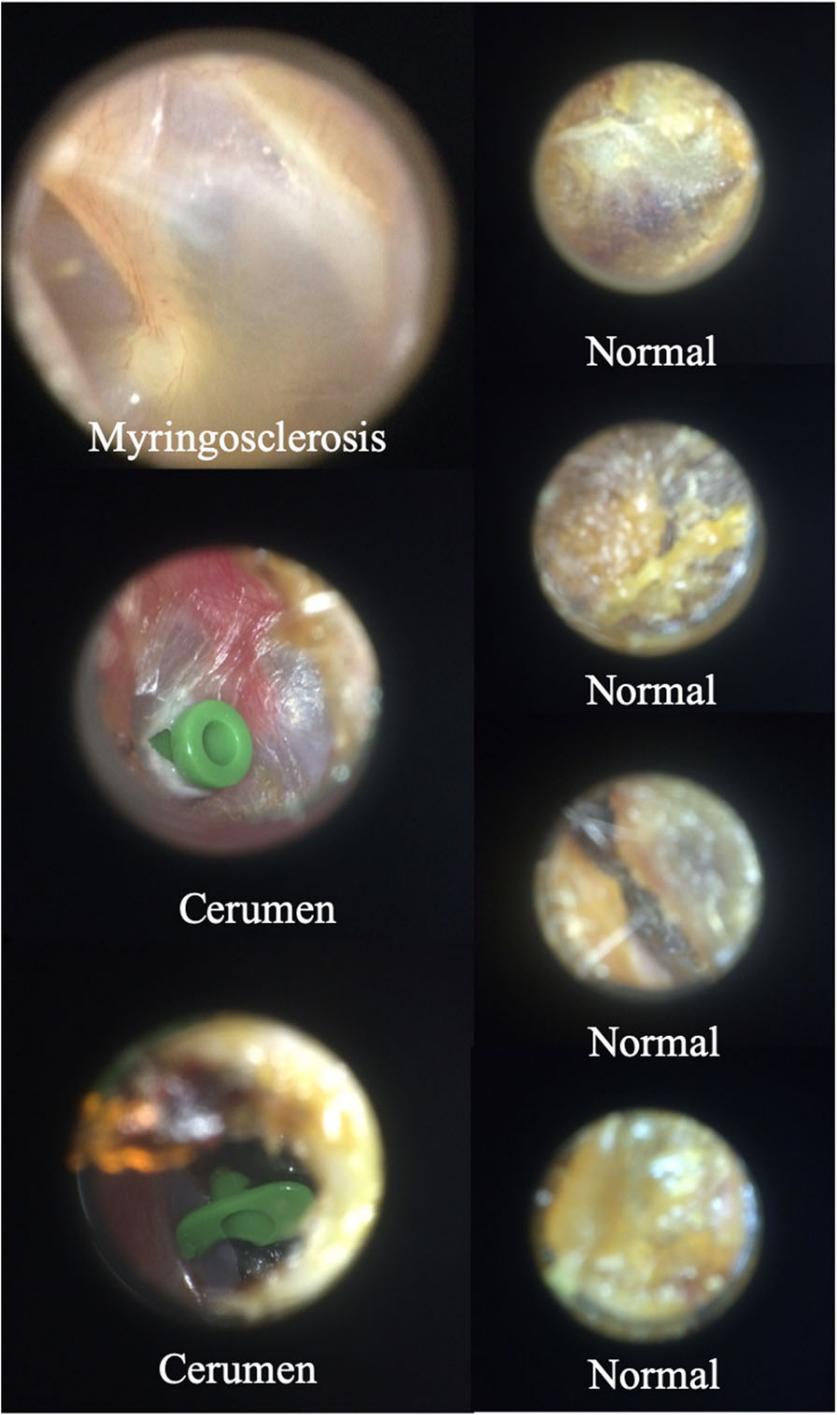
Misclassifications made by neural network (incorrect diagnosis on image)

Table 1 shows the performance of the algorithm when differentiating between normal tympanic membrane, cerumen impaction, and tympanostomy tube categories. The overall accuracy was 84.4% for these three diagnoses. There were 7 misclassifications overall, which can be seen in Fig. 2. The misclassifications for each diagnosis were consistent within each diagnostic category: two tympanostomy tube images were misclassified as cerumen impactions, four cerumen impactions were misclassified as normal, and one normal TM was misclassified as having myringosclerosis.

## Discussion

This work presents a first prototype implementation of a convolutional neural network for the multi-level classification of prominent ear conditions such as having a tympanostomy tube or cerumen impaction compared to a completely healthy ear. In detail, this deep neural network framework achieves an accuracy of 93.3, 86.7, 73.3% for the non-diseased ear, tympanostomy tube, and cerumen impaction categories, respectively. Figure 2 shows the images misclassified by the algorithm, which are clearly misclassified, even to the untrained eye. Though attempts to deconstruct why a machine learning algorithm mislabels data can only be speculative, doing so can help improve the quality of subsequent training data, while improving our understanding of how these algorithms work. For example, both images of ear tubes were misclassified as cerumen impactions. Certainly, in these images, there is a clinically insignificant amount of cerumen around the periphery of the external auditory canal that may have been detected by the algorithm. Within this context, it should also be pointed out that all images were converted to gray-scale images prior to CNN training and testing to prevent overfitting based on color information. Thus, the bright green color of the tympanostomy tube is not taken properly into account for the classification. With more datasets becoming available, this limitation can be addressed by using the full color information for CNN training and testing. Figure 2 shows the normal tympanic membrane that was misclassified as having myringosclerosis, which may have been due to the bright white bone of the scutum that was clearly visible through the ear drum in the posterior superior quadrant. The fact that all four cerumen impactions were misclassified as normal suggests that the algorithm is identifying features of wax that are associated with a non-diseased tympanic membrane, perhaps relating to features such as the surface topography. A multilabel classifier trained using a larger training dataset may have led to better classification results.

A machine learning approach for otoscopic diagnosis, as with any computer vision problem, requires a large number of images to reach optimal performance. This study was limited by the number of available images, so that image augmentation techniques such as mirroring and rotation were necessary to boost the number of images per category. However, augmentation alone does not provide the same value as a truly larger database for model optimization. While the testing images that where used for the validation of the approach were never part of the model training steps, the relatively small number of training cases seems to be the primary hindrance to higher accuracy levels, especially by making use of the full color information. Keeping in mind that our training sets were rather small, in order to avoid an overfitted network, we deliberately chose a smaller network, to avoid network memorization. Furthermore, we applied strong regularization methods such as dropout, batch normalization, and early stopping. Ultimately, various network parameters such as validation and training loss were consistently monitored to check for overfitting.

Only images from the same source were deliberately utilized in this study to avoid potential complications associated with the inclusion of multisource datasets. In detail, multisource images might have different image data attributes, such as lighting level, wavelength, aspect ratio, image resolution, and the size of otoscope tip. The diagnostic accuracy would likely be greatly decreased if multiple different imaging platforms were used for this study, especially if one diagnostic category was disproportionately recorded using a specific imaging modality leading to a systematic bias. For example, if a higher resolution camera such as a newer smart phone is used consistently in a tertiary otology clinic, with general ENT clinics using an older smart phone model, the algorithm might pick up on features associated with image resolution and inappropriately associate a disease diagnosis with high image resolution.

Distinguishing between wax impaction, an ear tube, and a normal ear is an easy task for any medical professional. Thus, the algorithm in its current form cannot supplant this basic physical exam skill. Our initial results, however, demonstrate that with a large enough dataset, computer vision could be successfully applied to support otoscopic diagnosis. Other authors, such as the Autoscope group, have leveraged machine learning approaches in combination with explicitly programmed feature extraction to expand the diagnostic capabilities of their algorithm to include multiple otologic diagnoses [11]. A collaborative, multi-institutional approach to obtaining high-quality images is necessary, and we welcome any individuals or groups that would like to work with us to accomplish this task. The 724 image otoscopy database generated for this work will be expanded with more diagnoses and images added over time. An open access selection of these images is available online for teaching and research purposes [16].

## Conclusion

We have built and implemented a first screening prototype software tool for otologic disease using a deep convolutional neural network. Our algorithm, while still rudimentary and inaccurate, is capable of identifying and differentiating between normal tympanic membranes, tympanostomy tubes, and cerumen impactions. Diagnostic performance on these and other otologic pathologies will improve as more images are obtained and incorporated into the algorithm training database. We hope that this algorithm, once adequately trained, could be utilized for telemedicine and global health applications where access to tertiary care level otolaryngology services is limited. Ideally, a low-cost and robust standalone image-capable otoscope with integrated software could be distributed to primary care providers, allowing them to use this framework as a diagnostic adjunct. This would improve diagnostic accuracy and would facilitate appropriate and timely referral patterns. In regions where access to ENT services is limited, an accurate automated diagnostic tool would represent a significant improvement in quality of care.

## Data Availability

Subset of otoscopic images available at www.entid.ca/atlas
